# Negative Effects of Stromal Neutrophils on T Cells Reduce Survival in Resectable Urothelial Carcinoma of the Bladder

**DOI:** 10.3389/fimmu.2022.827457

**Published:** 2022-03-21

**Authors:** Meihua Yang, Bo Wang, Weibin Hou, Hao Yu, Bingkun Zhou, Wenlong Zhong, Zhuowei Liu, Jinqing Li, Hong Zeng, Cheng Liu, Haide Qin, Tianxin Lin, Jian Huang

**Affiliations:** ^1^ Guangdong Provincial Key Laboratory of Malignant Tumor Epigenetics and Gene Regulation, Sun Yat-Sen Memorial Hospital, Sun Yat-Sen University, Guangzhou, China; ^2^ Department of Urology, Sun Yat-sen Memorial Hospital, Sun Yat-sen (Zhongshan) University, Guangzhou, China; ^3^ Department of Urology, Cancer Center, Sun Yat-sen (Zhongshan) University, Guangzhou, China; ^4^ Department of Pathology, Sun Yat-sen Memorial Hospital, Sun Yat-sen (Zhongshan) University, Guangzhou, China

**Keywords:** neutrophils, T cells, CD8, urothelial carcinoma of the bladder (UCB), survival

## Abstract

Urothelial carcinoma of the bladder (UCB) is a major type of bladder cancer with a distinct tumor microenvironment (TME). Although neutrophils are the main component of myeloid cells in the TME, the clinical significance and function of the neutrophils remain unclear in UCB. Here, we observed CD66b+ neutrophils were predominantly enriched in the stroma of UCB tissues and their levels emerged as an independent prognostic factor for overall survival (*P* = 0.006, n = 237), and were positively associated with age (*P* = 0.033), tumor stage (*P* < 0.0001), nodal metastasis (*P* = 0.045), and histological grade (*P* < 0.0001). Furthermore, we found that CD66b^+^ neutrophils were frequently co-localized with CD4^+^ T cells (R=0.35, *P* = 0.0067), CD8^+^ T cells (R=0.52, *P*<0.0001) and Cleaved Caspase-3^+^ apoptosis cells (R=0.44, *P* = 0.0007) in the stroma of UCB tissue. In addition, better effects of T cells on patients’ survival were markedly reduced by neutrophils and T cells co-infiltration. Moreover, we confirmed bladder tumor cell supernatant treated neutrophils suppressed T cell proliferation and activation, and promoted T cell apoptosis through GM-CSF induced PD-L1 *in vitro*. The expression of PD-L1 by neutrophils was also detected in fresh UCB tissues by using flow cytometric analysis. These data suggested that stromal CD66b+ neutrophils may potentially represent a reliable marker of poor prognosis for UCB patients, and neutrophils might play an immunosuppressive role on T cell immunity partially *via* the expression of PD-L1.

## Introduction

Mortality rates of urothelial carcinoma of the bladder (UCB) have declined in recent years partly due to the advents in immunotherapy, neoadjuvant therapy and targeted therapy (1–3), but only a subset of patients could benefit from current immunotherapy strategies (4); and the immunosuppressive microenvironment is still recognized as a significant barrier to effective treatment. UCB enriched in CD8+ T cells and associated with a poor clinical outcome exhibit abundant infiltration with dysfunctional macrophages, and a high expression of immune checkpoints and their ligands, which are mainly distributed in the tumor stroma rather than infiltrated into the tumor islets (5–7). Recently, neutrophils have emerged as similarly important contributors of the tumor stroma, and they are found at highly variable numbers within the tumor, depending on the cancer type (8).

Although neutrophils are the most abundant circulating leukocytes, the role of neutrophils in cancer has long been a matter of debate (9–11). Mature neutrophils express high levels of the chemokine receptors, CXCR1 and CXCR2. Their ligands, including CXCL1, CXCL2, CXCL3, CXCL5, and CXCL8 are frequently upregulated in tumors, which are important for neutrophil recruitment (12). In addition, tumor-associated neutrophils (TANs) can be activated *in situ* and the expression of CXCR1 and CXCR2 is downregulated on TANs (13, 14). Earlier studies have suggested that TANs have antitumor properties through direct cytotoxicity towards tumor cells (15). In contrast, emerging evidence indicates that TANs can promote tumor progression by stimulating angiogenesis and tumor cell migration (16–19). The prognostic and predictive power of TAN is associated with highly variable, and positive (early-stage lung cancer), negative (renal cancer), or controversial (colorectal carcinoma) correlations with the patient outcome in relevant studies (20–23). The markers used to identify TANs, including CD66b and myeloperoxidase (MPO), may explain these discrepancies, since the expression of these markers on neutrophils may vary in different tumor microenvironments (24). In UCB patients, early studies revealed neutrophils to be the main subset of cells found in the urine immediately following bacillus Calmette-Guerin (BCG) immunotherapy (25). Neutrophil infiltration in benign nodal tissue has been suggested to be associated with poor clinical outcome in patients with muscle-invasive bladder cancer (26). However, the clinical significance of TANs in UCB remains unclear and the underlying functional mechanisms have yet to be elucidated.

Herein, we analyzed the prognostic significance of both CD66b^+^ neutrophils in 237 cases of resectable UCB. We examined the intratumoral regions (INT) and stromal regions (ST) of the tumor tissues. In addition, we comparatively evaluated the neutrophil phenotypes from the tumor tissues and peripheral blood of UCB patients. Specifically, the distribution and prognostic relevance of the association of neutrophils with effector T cell infiltration in the UCB microenvironment was explored. Finally, the ability of the neutrophils to promote T cell apoptosis and inhibit its proliferation and activation partly in a PD-L1-dependent fashion was addressed.

## Materials and Methods

### Patients and Specimens

Tumor or blood samples were obtained from untreated patients who underwent a transurethral resection or cystectomy with pathologically confirmed UCB. No patients had metastasis and received preoperative anticancer therapies. Of these, formalin-fixed paraffin-embedded (FFPE) samples were enrolled from 237 patients between May 2003 and December 2009 at the Sun Yat-Sen University Cancer Center (Group 1, **Additional File 1: Table S1**) for survival analysis, which were divided into low or high groups based on the median frequencies of CD66b^+^ cells in intratumor (median=0 cell/field; n = 194 and 43, respectively) and stroma (median=5 cell/field; n = 121 and 116, respectively). During the follow-up for 237 patients (Group 1), 18 patients had distant metastasis, 79 patients had recurrence. FFPE tissues collected from 57 patients between June 2015 and January 2016 at Sun Yat-sen memorial hospital (Group 2, **Additional File 1: Table S2**) were used to evaluate neutrophil and T cell-associated functional markers by immunohistochemistry. All tumors were graded according to the World Health Organization 2014 classification and staged according to the TNM classification (8^th^ edition, 2016). To evaluate the prognostic value in subgroups, the 237 UCB patients (Group 1) were divided into non-muscle-invasive bladder cancer (NMIBC; n =171) or muscle-invasive bladder cancer (MIBC; n =66) subgroups based on the tumor stage and into low (n =145) or high (n =92) grade subgroups based on histological grade (**Additional File 1: Table S1**). The use of clinical information was approved by the local ethical authorities. Moreover, The Cancer Genome Atlas (TCGA) data of 408 bladder cancer patients was obtained from the TCGA database and analyzed by the CIBERSORT method to obtain the neutrophil representation in RNA level from bulk tumor transcriptomes ^27.^

### Immunohistochemistry and Immunofluorescence

FFPE tissues from study Groups 1 and 2 were cut into 5-μm sections, and processed for immunohistochemistry as previously described (6, 27, 28). Briefly, after an incubation with antibodies (Abs) specific for CD66b, CD4, CD8, PD1, Cleaved Caspase-3, or control Abs (**Additional File 1: Table S3**), the adjacent sections were developed with peroxidase-conjugated secondary Abs and stained with peroxidase and 3,3′-diaminobenzidine tetrahydrochloride in an Envision System (Dako). Multiplexed fluorescent immunohistochemistry was performed using the Tyramide Signal Amplification method with an Opal IHC kit (PerkinElmer), and FFPE tissue sections were processed according to the standard IHC protocol described above. The primary Abs were anti-human CD66b, CD4, CD8, MPO, or PD-L1, followed by an incubation with appropriate peroxidase-conjugated secondary Abs (Dako). Detection was performed with an incubation with FITC-, Cy3-, or Cy5-labeled Tyramide. The slides were counterstained with DAPI (Sigma Aldrich). The Zeiss LSM710 system with ZEN software (Zeiss, Oberkochen, Germany) was used to capture images of the stained slides and perform image analysis.

### Evaluation of Immunohistochemical Variables

Tissue sections from Groups 1 and 2 were analyzed by two independent pathologists who were blinded to both the clinicopathological and survival data. The density of CD66b^+^, CD4^+^, CD8^+^, PD1^+^, or Cleaved Caspase-3^+^ stromal cells in the INT and/or ST regions were screened at a low power field (100× magnification) and five of the most representative high-power fields were selected at 400× magnification (0.07 mm^2^ per field) for each area of all specimens. The total number of these cell densities in each area were counted manually. Data are expressed as the means ± SEM to indicate the number of cells per field.

### Preparation of a Single-Cell Suspension From UCB Tissues

A single-cell suspension from UCB tissues was obtained as previously described (20). Briefly, surgically removed fresh UCB tissues were trimmed, sliced into small pieces, and digested. The dissociated cells were filtered through a 70-µM nylon cell strainer (BD Falcon). After filtration, red blood cells were lysed, and the remaining cells were washed and re-suspended. For further detail, see the **Supplementary Materials**.

### Tumor Cell Line and TSN Preparation

See **Supplementary Materials**.

### Neutrophil and Lymphocyte Isolation

Peripheral blood mononuclear cells (PBMCs) from buffy coats derived from healthy donors were isolated by Ficoll density gradient centrifugation. CD3^+^ T cells were purified from PBMCs using a Pan T cell isolation kit (Miltenyi Biotec). After the lysis of red blood cells, peripheral blood neutrophils (PBNs) were further enriched by positively removing contaminating cells using an EasySep Human Neutrophil Enrichment Kit (Stemcell Technologies). The purity of the isolated PBNs was evaluated by flow cytometry *via* staining for neutrophils/myeloid markers CD66b, CD15, CD62L, and CD11b in the cells used in functional assays. The sorted cells were not used unless their viability was determined to be > 90% and their purity was > 95%. In some experiments, the PBNs were cultured in complete culture medium in the presence of 20% TSN for 24 h to obtain TSN-treated neutrophils (TTNs) to mimic TANs in UCB tissues.

### Flow Cytometry

Flow cytometric analysis was performed according to standard protocols. Cell suspensions from the tissues and peripheral blood of UCB patients, culture neutrophils, or T cells, were stained *in vitro* with fluorochrome-conjugated Abs and matched-isotype control Abs (**Additional File 1: Table S3**). The stained cells were subsequently analyzed by multicolor flow cytometry. For additional detail, see the **Supplementary Materials**.

### Neutrophil-T Cell Co-Culture System

PBNs and matched TTNs were co-cultured with T cells from the autologous peripheral blood of healthy donors to study the function of neutrophils on T cell proliferation, activation, and apoptosis. Briefly, bead-purified peripheral CD3^+^ T cells were labeled with carboxyfluorescein succinimidyl ester (CFSE) and co-cultured with autologous PBNs or TTNs at a 1:1 ratio in CD3-coated plates for three days. In several experiments, a PD-L1 blocking Ab (Thermo Fisher Scientific, eBioscience) was added to the co-cultures. To assess cellular proliferation and activation, the CFSE and IFN-γ signals were analyzed by flow cytometry on gated CD4^+^ or CD8^+^ T cells. To assess apoptosis, T cells were stained with an AnnexinV-PI staining kit according to the manufacturer’s instructions (BestBio). For additional details see the **Supplementary Materials**.

### Statistical Analysis

Statistical analyses were performed using SPSS 16.0 software (SPSS Inc., Chicago, IL, USA). The statistical significance of the differences between groups was determined using a Wilcoxon signed-rank test. Correlations between parameters were assessed using a Pearson correlation analysis and linear regression analysis, as appropriate. The cumulative overall survival (OS) and recurrence-free survival (RFS) time was calculated using the Kaplan-Meier method and analyzed with a log-rank test. A multivariate Cox proportional hazards model was used to estimate the adjusted hazard ratios and 95% CIs, and identify independent prognostic factors. For the categorical analyses, the median value was used as a cut-off to dichotomize the continuous variables (for clinical applications). For TCGA data, the associations between clinical outcomes and TANs were analyzed with R software package, version 3.5.1 (The R Foundation for Statistical Computing, http://www.r-project.org/). For such comparisons, two-tailed P-values < 0.05 were considered to indicate statistically significant differences.

## Results

### Phenotypes and Prognostic Significance of TANs in Human UCB Tissues

**Figure 1:**
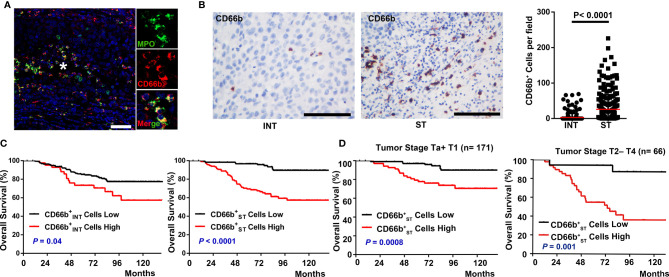
Spatial distribution of neutrophils and prognostic significance of their density with overall survival in UCB patients. **(A)** Paraffin-embedded UCB sections were subjected to two-color immunofluorescence staining for CD66b (red) and MPO (green). * marked the magnified field. Scale bar: 20 μm. **(B)** Distribution of CD66b^+^ in the intratumoral (INT) and stromal (ST) regions of UCB tissues (n = 237). Scale bar: 100 μm. Cell numbers were calculated as the cell count per 400× field. Data are expressed as the means ± SEM. **(C)** Cumulative overall survival curves of the patients. Patients were divided into two groups according to the median number of CD66b^+^ cells per 400× fields, including ${CD66b}_{\text{INT}}^{+}$ cells in INT regions ( ${CD66b}_{\text{INT}}^{+}$ cells, median = 0 cell/field), CD66b^+^ cells in ST regions ( ${CD66b}_{\text{ST}}^{+}$ cells, median = 5 cell/field). **(D)** Kaplan-Meier overall survival curves stratified by tumor stage. NMIBC (non-muscle-invasive bladder cancer, tumor stage Ta+T1, *n* = 171) and MIBC (muscle-invasive bladder cancer, tumor stage T2-T4, *n* = 66); Kaplan-Meier survival estimates and log-rank tests were used to analyze the prognostic significance of these cells. Black lines, low group; red lines, high group.

Neutrophils exhibit diverse phenotypes during inflammation and tumor pathogenesis (29). To precisely identify TANs in UCB, we used two complementary approaches, immunofluorescence/immunohistochemistry of cells within the tumor tissue presented *in situ*. Using two-color immunofluorescence analyses, we observed variability regarding the co-localization of CD66b and MPO between patients (**Figure 1A**), indicating the heterogeneous population of neutrophils in cancer.

Previous studies have shown that neutrophils can be detected in different locations within and around a tumor (13, 24). We found that the frequencies of both CD66b^+^ cells were significantly higher in the ST regions (26.06 ± 2.68cells/field, **Figure 1B**) than in the corresponding INT regions of the UCB tissues (3.2 ± 0.72 cells/field, **Figure 1B**). To investigate the association between diverse neutrophil phenotypes with UCB progression, we divided 237 UCB patients (Group 1) into two groups based on the median frequencies of CD66b^+^ cells in intratumor and stroma respectively (**Additional File 1: Table S4**). A marked negative association was found between the OS and the density of ${CD66b}_{\text{INT}}^{+}$ cells (*P* = 0.04, **Figure 1C**), ${CD66b}_{\text{ST}}^{+}$ cells (*P* < 0.0001, **Figure 1C**). The five-year OS rate was 73% above the median compared to 85% below the median for ${CD66b}_{\text{INT}}^{+}$ cells, 69% vs. 97% for ${CD66b}_{\text{ST}}^{+}$ cells. Moreover, consistent with previous study (30), we applied the CIBERSORT algorithm to the bladder cancer data in TCGA and found the abundance of TANs was also negatively correlated to OS (*P* = 0.0034, **Additional File 1: Figure S1A**). However, the presence of CD66b^+^ cells was not associated with any prognostic significance for RFS (all *P* > 0.05, **Additional File 1: Figure S1B, C**). When patients in Group 1 were divided into ≤60 and >60 years old groups, age was an independent predictor for OS using multivariate analysis (HR, 3.46; *P* < 0.0001; **Table 1**). A multivariate analysis revealed that the density of CD66b^+^ ST cells rather than CD66b^+^ INT cells was an independent prognostic factor for OS (HR, 3.477; *P* = 0.006; **Table 1**). Interestingly, the density of ${CD66b}_{\text{ST}}^{+}$ cells was positively associated with age, tumor stage, nodal metastasis, and histological grade, all of which are indicators for poor prognosis (**Additional File 1: Table S6**), thus we further evaluated the prognostic value of ${CD66b}_{\text{ST}}^{+}$ cells in subgroups of patients stratified by tumor stage (NMIBC: *P* = 0.0008, MIBC: *P* = 0.001; **Figure 1D**) and histological grade (**Additional File 1: Figure S2**). High density of ${CD66b}_{\text{ST}}^{+}$ cells could be a new prognostic value for a shorter OS.

**Table 1:** Univariate and Multivariate Analysis of the Factors Associated with Overall Survival.

Variable	Univariate			Multivariate		
	HR	95% CI	P	HR	95% CI	P
Age, years (>60/≤60)	3.53	1.943–6.413	**3.5×10^–5^**	3.46	1.882–6.363	**<0.0001**
Gender (female/male)	0.694	0.276–1.741	0.436			NA
Tumor size (>3 cm/≤3 cm)	1.626	0.896–2.951	0.11			NA
Multifocality (Multifocal/Unifocal)	0.922	0.502–1.695	0.794			NA
Tumor stage (T2–T4/Ta–T1)	3.429	2.009–5.854	**6.3×10** ^–6^	2.214	1.163–4.214	**0.016**
Nodal status (N1–N2/N0)	6.644	2.933–15.051	**5.7×10** ^–6^	3.931	1.601–9.65	**0.003**
Histological grade (High/Low)	2.113	1.236–3.612	**0.006**	1.003	0.531–1.894	0.992
${CD66b}_{\text{INT}}^{+}$ Cells (High/Low)	1.954	1.077–3.546	**0.028**	0.974	0.488–1.944	0.941
${CD66b}_{\text{ST}}^{+}$ Cells (High/Low)	5.715	2.871–11.374	**6.9×10^–7^**	3.477	1.434–8.428	**0.006**

${CD66b}_{\text{INT}}^{+}$ Neutrophils, CD66b^+^ Neutrophils in introtumoral regions; ${CD66b}_{\text{ST}}^{+}$ Neutrophils, CD66b^+^ Neutrophils in stromal regions. UCB, urothelial cell carcinoma of the bladder. HR, hazard ratio; CI, confidence interval; NA, not applicable.

Univariate and multivariate analysis. Cox proportional hazards regression model. Variables associated with survival by univariate analyses were adopted as covariates in multivariate analyses. Significant P-values are shown in bold font. HR > 1, risk for death increased; HR < 1, risk for death reduced.

### Association Between TANs and T Cell Infiltration in ST of UCB Tissues

**Figure 2:**
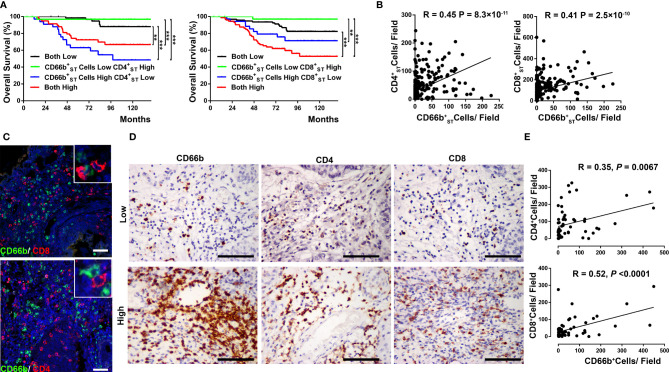
Co-localization of stromal neutrophils and T cells associated with Overall survival. **(A)** Kaplan-Meier overall survival (OS) curves were designed according to high- and low-density of combined stromal CD66b^+^ neutrophil and CD4^+^ T cell infiltration or combined stromal CD66b^+^ neutrophil and CD8^+^ T cell infiltration. Patients with high levels of CD4^+^/CD8^+^ T cell infiltration alone had a significantly better OS than patients with high levels of combined CD4^+^/CD8^+^ T cell and neutrophil infiltration. *P*-values were determined using a log-rank test. ** *P* < 0.001; *** *P* < 0.0001. **(B)** Positive associations between the number of ${CD66}_{\text{ST}}^{+}$ cells and ${CD4}_{\text{ST}}^{+}$ cells, ${CD66}_{\text{ST}}^{+}$ cells and ${CD8}_{\text{ST}}^{+}$ cells (n = 237). **(C)** Representative pictures showing the co-localization of neutrophil (CD66b, green) and T cell markers (CD4 and CD8, red) in the stromal (ST) regions of UCB tissues. **(D, E)** Consecutive sections were used for the immunohistochemical identification of stromal CD66b^+^ cells, as well as CD4^+^ T cells and CD8^+^ T cells in UCB tissues (n= 57). P < 0.05 was considered significant difference (Spearman’s rank correlation coefficient test). Representative examples of tumors with either low (top) or high (bottom) expression of neutrophil (CD66b), T cells (CD4 and CD8) markers are shown. Scale bar: 100 μm.

Based on our initial findings, we considered that the mechanism of action underlying the unfavorable prognostic significance of TANs might be, as former studies reported, related to their ability to suppress T cell immunity in UCB. Firstly, we investigate the prognostic significance of combined stromal CD66b^+^ cell and T cell infiltration in UCB patients. Using the median number as the cut-off, patients were classified into four groups based on ${CD66b}_{\text{ST}}^{+}$ cell and ${CD4}_{\text{ST}}^{+}$ T cell infiltration or ${CD66b}_{\text{ST}}^{+}$ cell and ${CD8}_{\text{ST}}^{+}$ T cell infiltration (**Figure 2A**, respectively: 1) low ${CD66b}_{\text{ST}}^{+}$ cells and low T cells; 2) low ${CD66b}_{\text{ST}}^{+}$ cells and high T cells; 3) high ${CD66b}_{\text{ST}}^{+}$ and low T cells; and 4) high ${CD66b}_{\text{ST}}^{+}$ and high T cells). Significant differences in OS were found among the four groups based on the two combinations (**Figure 2A**). Notably, the present results showed that UCB infiltrated by low ${CD66b}_{\text{ST}}^{+}$ cells and high T cells was associated with the most favorable prognosis, whereas a high infiltration of ${CD66b}_{\text{ST}}^{+}$ cells could reverse the beneficial effects of both CD4^+^ (**Additional File 1: Table S6**) and CD8^+^ T cell (**Additional File 1: Table S7**) responses in the tumor stroma, which is at least in part due to the immunosuppressive effects of tumor-infiltrating neutrophils on T cells.

In Group 1 UCB tissues, we also found that the density of stromal CD66b^+^ cells was positively associated with both CD4^+^ and CD8^+^ T cells in the same area (respectively: R=0.45, *P* < 0.0001; R=0.41, *P* < 0.0001; **Figure 2B**). Furthermore, using two-color immunofluorescence analyses, we observed that CD66b^+^ cells are frequently colocalized with both CD4^+^ T cells and CD8^+^ T cells within the ST of UCB tissues (**Figure 2C**). We also confirmed these results in Group 2 UCB tissues from a prospective cohort of 57 UCB patients (CD4, R=0.35, *P*= 0.0067; CD8, R=0.52, *P* < 0.0001; **Figures 2D, E**). These results suggested that neutrophils might play a role on T cell effector function in UCB tumor progression within the ST area.

### Negative Effects of TANs on T Cell Proliferation, Activation, and Apoptosis

The co-localization of TANs and T cells in the stroma of UCB tissues suggests that neutrophils might promote tumor progression by modulating T cell immunity. Using multicolor flow cytometry of cells harvested from the human fresh UCB tissues, it revealed that CXCR1, CXCR2, and CD62L are expressed to a lower extent in CD11b^+^CD66b^+^ TANs compared with autologous PBNs (**Additional File 1: Figure S3A**). To mimic TANs, we exposed PBNs from healthy donors to TSN collected from the T24 bladder cancer cell line, defined as TTNs. TTNs was observed to downregulate the expression of CXCR1, CXCR2, and CD62L on the surface of PBNs, which is consistent with the expression of these molecules on TANs (**Additional File 1; Figure S3A, B**).

**Figure 3:**
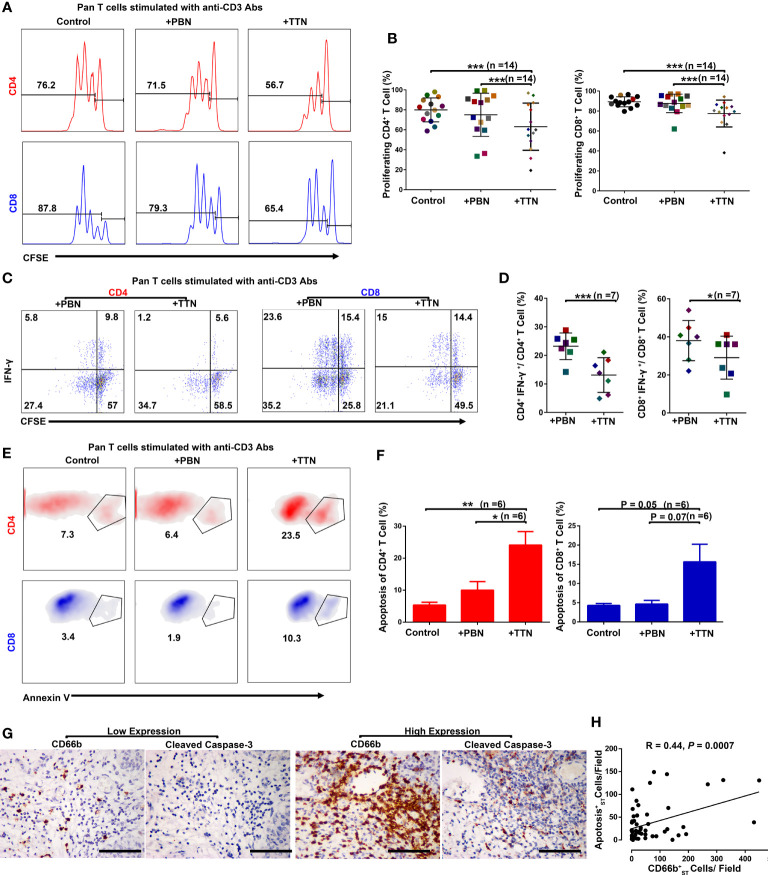
T cell proliferation, apoptosis, and activation in the presence of PBNs or TTNs. In all experiments, autologous T cells were purified from PBMCs, stimulated with plate-bound anti-CD3/CD28 Abs, and were cultured alone or with PBNs or the tumor cell line T24 supernatant-treated PBNs (TTNs) at a 1:1 ratio for five days. **(A, B)** Flow cytometric analysis of autologous CD4^+^ T cells and CD8^+^ T cells proliferation in the presence of PBNs or TTNs (n = 14). **(C, D)** Flow cytometric analysis of autologous CD4^+^ T cells and CD8^+^ T cells IFN-γ production in the presence of PBNs or TTNs (n = 7). **(E, F)** Flow cytometric analysis of autologous CD4^+^ T cells and CD8^+^ T cells apoptosis in the presence of PBNs or TTNs (n = 6). Numbers on histograms or a density plot represented the percentage of T cell proliferation or apoptosis, respectively **(A, E)**. Numbers in quadrants indicate the percentage of cells in each quadrant **(C)**. Error bars represent the mean ± SEM. P < 0.05 was considered a significant difference (Student’s *t-*test, paired parametric test). **(G, H)** Consecutive sections were used for the immunohistochemical identification of stromal CD66b^+^ cells and apoptosis makers (Cleaved Caspase-3) in UCB tissues (n= 57). P < 0.05 was considered significant difference (Spearman’s rank correlation coefficient test). Representative examples of tumors with either low (top) or high (bottom) expression of neutrophil (CD66b), apoptosis (Cleaved Caspase-3) markers are shown. Scale bar: 100 μm. * *P* < 0.05; ** *P* < 0.001; *** *P* < 0.0001.

To test the hypothesis that TTNs could recapitulate the ability of TANs to suppress T cell proliferation, PBNs and TTNs were co-cultured with autologous purified peripheral CD3^+^ T cells from healthy donors that had been labeled with CFSE and stimulated with plate-bound anti-CD3 Abs for three days. The results showed that the proliferation and apoptosis of the stimulated T cells were not altered by exposure to PBNs. However, when the stimulated CD3^+^ T cells were co-cultured with TTNs, the proliferation of CD4^+^ and CD8^+^ cells were markedly inhibited (**Figures 3A, B**), and TTNs could suppress the proportion of IFN-γ-expressing CD4^+^ and CD8^+^ T cells following *ex vivo* PMA/ionomycin stimulation (**Figures 3C, D**). Moreover, TTNs also significantly triggered the apoptosis of CD4^+^ and CD8^+^ cells in this co-culture system (**Figures 3E, F**). In Group 2 UCB tissues (n = 57), the density of stromal CD66b^+^ cells are strongly positively associated Cleaved Caspase-3^+^ apoptosis cells (R=0.44, *P*=0.0007; **Figures 3G, H**). These data indicate that bladder cancer associated TANs have negative effects on T cell proliferation, activation and apoptosis.

### TANs Suppress T Cell Immunity *via* PD-L1

**Figure 4:**
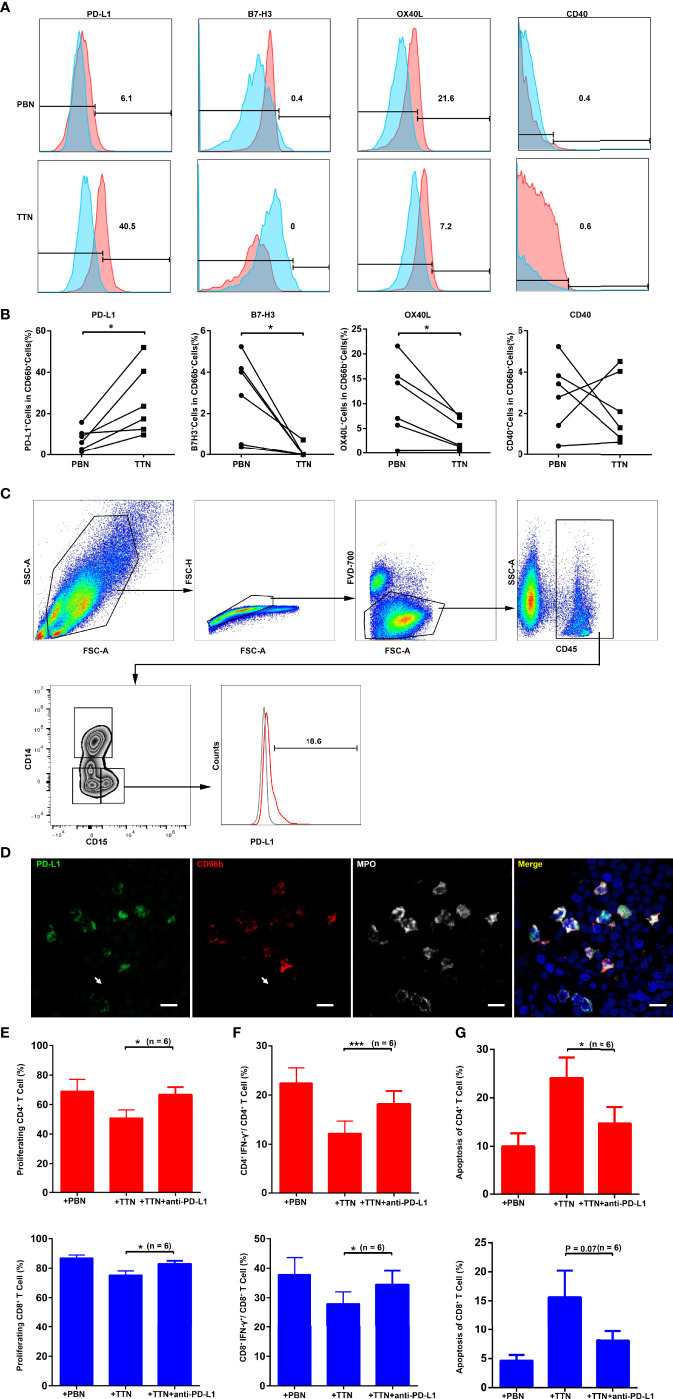
The expression of PD-L1 on TTN and its role in the suppression of T cell immunity. **(A)** The expression of co-suppressive molecules (PD-L1 and B7-H3) and co-stimulatory molecules (OX40L and CD40) were analyzed by flow cytometry on gated live CD11b^+^CD66b^+^ PBNs (top) and tumor cell line T24 supernatant-treated PBNs (TTNs, bottom). Results from one out of six representative experiments are shown. **(B)** The panel summarizes the change in the percentage of the indicated markers on autologous PBNs and TTNs. **(C)** The expression of PD-L1 were analyzed by flow cytometry on gated CD45^+^CD15^+^ TANs in human fresh UCB tissues. (Gray: Isotype; Red: TANs). **(D)** Representative pictures showing the co-expression of PD-L1 with CD66b and MPO on tumor-infiltrating neutrophils in UCB tissues. **(E–G)** The efficacy of a PD-L1 blocking Ab in ablating the effect of TTNs on T cell proliferation **(E)**, IFN-γ production **(F)** and apoptosis **(G)**. Data shown are from six independent experiments in **E–G**, respectively. Error bars represent the mean ± SEM. P < 0.05 was considered a significant difference (Student’s *t-*test, paired parametric test). * *P* < 0.05; *** *P* < 0.0001.

Since changes in immunosuppressive phenotypes are correlated with leukocyte activation, we next quantified the expression of co-stimulatory/co-inhibitory molecules on PBNs and TTNs by flow cytometry. Compared with PBNs, PD-L1 expression was markedly upregulated on TTNs, whereas OX40L expression was significantly downregulated on TTNs (**Figure 4A, B**). Moreover, both PBNs and TTNs express low levels of B7-H3 and CD40.

By using multicolor flow cytometry of the human fresh UCB tissues, we find CD45^+^CD15^+^ TANs express PD-L1 (**Figure 4C**). More importantly, using *in situ* triple color immunofluorescence, we observed the co-expression of PD-L1 with CD66b and MPO on tumor-infiltrating neutrophils in UCB tissues (**Figure 4D**). In addition, we found the density of stromal CD66b^+^ cells are strongly positively associated PD 1^+^ cells in 57 UCB tissues (Group 2) (R=0.47, *P* = 0.0002; **Additional File 1; Figure S4A, B**).

To further elucidate whether PD-L1 is involved in the TTN-mediated suppression of T cell immunity, we added a blocking Ab against PD-L1 in a TTN and CFSE-labeled activated autologous T cell co-culture system. The anti-PD-L1 Ab reinvigorated the strong suppressive effect of the TTNs on T cell proliferation (**Figure 4E**, **Additional File 1; Figure S4C**) and activation (**Figure 4F**, **Additional File 1; Figure S4E**), as well as reversed the promotion of TTNs on T cell apoptosis (**Figure 4G**, **Additional File 1; Figure S4D**).

### Tumor-Secreted Factor GM-CSF Triggered PD-L1 Expression on Neutrophils

**Figure 5:**
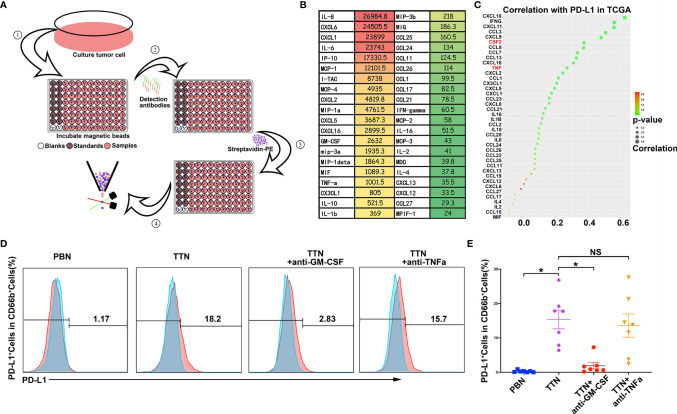
Tumor-secreted factor GM-CSF triggered PD-L1 expression on neutrophils. **(A)** The workflow of Bio-Plex Multiplex Immunoassays. **(B)**The heatmap of supernatant cytokines profile in the tumor cell line T24 cultured for 48 hours. The color represents the concentration (ng/ml) of cytokines. Green, low concentration; Red, high concentration; Yellow, middle concentration. **(C)** The dotplot showing the correlation between PD-L1and cytokines in the TCGA database. *P*-values were determined using Pearson’s correlation coefficient test. The color represents *P*-values Green, low *P*-values; Red, high *P*-values, the size represents correlation values. **(D, E)** The efficacy of blocking GM-CSF and TNFα in ablating the expression of PD-L1 on TTNs. **(D)** Representative experiments (n=7). **(E)** Summarizes the change in the percentage of PD-L1+ TTNs and add blockade antibody of GM-CSF and TNFα into tumor cell line T24 supernatant-treated TTNs. Error bars represent the mean ± SEM. * *P* < 0.05 was considered a significant difference, NS was no significance (Student’s t-test, paired parametric test).

Previous studies showed that GM-CSF and TNFα could be inducers of PD-L1 expression by many immune cells (31). We also found GM-CSF and TNFα were highly secreted by T24 bladder cancer cell line by Bio-Plex Multiplex Immunoassays (**Figures 5A, B**). Moreover, in TCGA dataset, the level of GM-CSF and TNFα have significantly positive association with PD-L1 expression in bladder tissues (**Figure 5C**). To further evaluate the potential role of tumor-secreted GM-CSF and TNFα on PD-L1 induction by neutrophils, neutralizing antibodies against GM-CSF and TNFα were separately added to the TSN/neutrophil co-culture system. The results showed that the expression of PD-L1 on TTNs was blockaded efficiently by neutralizing GM-CSF but not TNFα (**Figures 5D, E**).

## Discussion

The role of TANs in tumor immunology and their clinical significance in tumor tissues remains controversial (10, 24). In the present study, we demonstrated that the density of ${CD66b}_{\text{ST}}^{+}$ cells was associated with advanced disease and an independent predictor of poor OS in 237 UCB patients. Moreover, the number of ${CD66b}_{\text{ST}}^{+}$ cells was positively associated with the density of both ${CD4}_{\text{ST}}^{+}$ and ${CD8}_{\text{ST}}^{+}$ T cells. A combination OS analysis showed that ${CD66b}_{\text{ST}}^{+}$ cells markedly reduced the favorable prognosis of ${CD4}_{\text{ST}}^{+}$ and ${CD8}_{\text{ST}}^{+}$ T cells. Moreover, an *in vitro* functional study indicated that TTNs can inhibit T cell antitumor immunity partially through the PD-L1 pathway. Such neutrophil/T cell crosstalk supports the concept of a highly functional cooperation between the innate and adaptive immune responses in the tumor microenvironment.

Human tumor tissues can be anatomically classified into INT and ST regions, each of which has a distinct composition and functional properties (32, 33). Most studies have shown that INT neutrophils, as opposed to ST neutrophils, have a strong association with detrimental prognoses (e.g., renal cell carcinoma, melanoma, and lymphoma) (21, 34, 35). Nevertheless, the present study showed that both CD66b^+^ TANs are predominantly distributed in the ST compared to the INT. Although all of these cells were associated with an inferior OS in the univariate analysis, only ${CD66b}_{\text{ST}}^{+}$ TAN density has been identified as an independent prognosis factor for UCB patients, as previously observed in samples from hepatocellular carcinoma patients (36). Overall, tumor-infiltrating neutrophils were significantly negatively correlated with patient prognosis, which was confirmed by previous studies (30, 37). In addition, there studies also found a negative correlation with RFS, which was not found in our study. The different predictive value of TANs for OS and RFS may be partly due to the limited numbers of patients in our current cohort. Moreover, a correlation analysis showed that the ${CD66b}_{\text{ST}}^{+}$ TAN density was positively associated with tumor stage, nodal metastasis, and histological grade. In addition, our profiling of neutrophils within UCB confirms that they are phenotypically distinct from their peripheral counterparts, displaying low expression of the adhesion molecule, CD62L, and chemokine receptors, CXCR1 and CXCR2. These findings suggest that TANs in the stroma may represent a robust, stable, and reliable prognostic indicator for UCB.

Evidence has shown that TANs have the capacity to modulate other immune cells during tumor progression (10, 38). The apoptotic neutrophils can promote macrophages to differentiate into M2, which reduced the stimulation of allogeneic T cell proliferation. Our results indicate that TTNs co-cultured with autologous T cells can reduce T cell receptor-triggered T cell proliferation and activation, as well as promote T cell apoptosis. In addition, we observed the co-localization of TANs and CD4^+^ T cells, CD8^+^ T cells, and the apoptosis marker, cleaved caspase-3 in the stroma of UCB tissues. Moreover, a positive association was also found between the expression of chemokines that recruit neutrophils and T cells. These results suggest that the tumor microenvironment can simultaneously recruit neutrophils and T cells into the same location; however, TANs have a suppressive function on T cells. Notably, a combination OS analysis showed that high T cell but low CD66b^+^ cell infiltration in the stroma was characterized by a more favorable prognosis compared with other groups. In addition, a high level of neutrophil infiltration can significantly reduce a good prognosis based on the presence of T cells. Therefore, neutrophil-T cell interactions may reverse the antitumor effects of T cells or induce new pro-tumoral phenotypes and functions to support tumor progression.

Neutrophils can respond to diverse stimuli to support or inhibit T cell immunity in different types or stages of tumors (13, 24). Additionally, in response to CXCL8, neutrophils can produce arginase-1 to inhibit antitumor immunity (39). Moreover, tumor-derived GM-CSF can induce high PD-L1 expression on TANs, which exhibits a more profound suppressive role on T cell proliferation and IFN-γ production in human gastric cancer (40). Accordingly, the depletion of neutrophils in mouse lung tumors results in increased activation of CD8^+^ T cells and decreased tumor growth that can be mediated by TGFβ1 (41). However, during early-stage human lung cancer, TANs can instead promote T cell-mediated immunity through the costimulatory molecules, OX-40L and 4-1BBL, to enhance the proliferation and activation of T cells 41. Herein, we found that bladder cancer cell supernatants can induce high levels of PD-L1 expression on neutrophils and reduce the level of OX-40L expression. Furthermore, blocking PD-L1 can reverse the immune suppression mediated by TTN/T cell crosstalk. There was also a positive association observed between the TANs and PD-1^+^ T cells in the stroma. Our results further demonstrate the complexity of TAN/T cell crosstalk in UCB tissues and emphasize the importance of the PD-L1/PD1 pathway in tumor-related immunosuppression. Moreover, emerging studies had reported that neutrophils are closely correlated with the efficacy of ICIs in non-small cell lung cancer (NSCLC) (42) and the expression of IL-8 in serum reduced the efficacy of immunosuppressive agents *via* increased intertumoral neutrophils (43). The value of neutrophils to predict the outcome of ICIs for advancer MIBC need further investigate.

This study has several limitations. First, UCB can be further categorized into two distinct types: 1) NMIBC; and 2) MIBC (44) Although the CD66b^+^ TAN density was inversely associated with the progression of UCB and maintained its prognostic value in predicting a shorter OS stratified according to tumor stage, there are limited number of matched tissues from patients who progressed from NMIBC to MIBC. This lack of matched tissues restricts the value of longitudinal studies addressing the role of TANs in different stages of tumor progression. Second, although previous studies have shown that BCG installation can increase neutrophil infiltration to inhibit tumor progression, the presence of high TANs identified a subgroup of MIBC patients who appeared to benefit from adjuvant chemotherapy, suggesting that neutrophil functions can be modulated by different treatments (30). Notably, this present retrospective analysis only assessed patients with treatment-naïve UCB. Thus, we cannot compare our results with that of other studies of patients treated with BCG immunotherapy, chemotherapy, or radiotherapy. Finally, TANs generated from single-cell suspensions of fresh UCB tissues are present in low numbers due to a short life span or enzyme sensitivity, which prevents the routine performance of functional studies (24, 45). Our functional study used tumor supernatant-treated peripheral blood neutrophils from healthy donors to mimic TANs in tumor tissues. Although TTNs and TANs have similar phenotypes, the direct functions of TAN and T cell crosstalk *in situ* warrants further investigation.

In conclusion, by combining IHC, biomolecular analyses, and functional experiments, we have comprehensively documented the influence of TAN/T cell crosstalk on the establishment of the immune context associated with UCB. Although the clear mechanisms of the TAN-induced immunosuppressive function cannot be confirmed, our data suggest that TANs can revert the favorable prognosis of T cells in the stroma, partially *via* the PD-L1 pathway. Accordingly, we identified tumor infiltration by CD66b+ TANs in the stroma to be a robust poor prognostic biomarker for UCB patients, representing an additional example of clinically relevant interaction between stomal cells in the tumor microenvironment.

## Data Availability Statement

The original contributions presented in the study are included in the article/**Supplementary Material**. Further inquiries can be directed to the corresponding authors.

## Ethics Statement

Written informed consent was obtained from the individual(s), and minor(s)’ legal guardian/next of kin, for the publication of any potentially identifiable images or data included in this article.

## Author Contributions

All authors have contributed significantly, and that all authors are in agreement with the content of the manuscript. MY and BW designed the experiment, analyzed the data and wrote the manuscript. WH and HY performed the immunohistochemical staining. BZ and JL performed the flow cytometry. ZL, WZ, and HQ collected the samples and clinic data. HZ and CL evaluate the immunohistochemical makers of tissue sections. TL modified and revised the manuscript. JH supervised in the design of the study and finalized the manuscript. All authors contributed to the article and approved the submitted version.

## Funding

This study was supported by the National Natural Science Foundation of China (Grant No. 2018YFA0902803, 81572514, 81402106, 81702391, 81702523, 81772719, 81702525, 81772728, 81740119, 81825016 and 81961128027); Guangdong Science and Technology Development Fund (2017B020227007 and 2018B010109006); Science and Technology Program of Guangzhou (Grant No. 201806010024); National Natural Science Foundation of Guangdong (Grant No. 2015A030310122); Pearl River Nova Program of Guangzhou (201806010024); the Cultivation of Major Projects and Emerging, Interdisciplinary Fund, Sun Yat-Sen University (Grant No. 16ykjc18); Elite Young Scholars Program of Sun Yat-Sen Memorial Hospital (Grant No. J2016-106), Elite Young Scholars Development Program of Sun Yat-Sen Memorial Hospital to BW; Yat-Sen Scholarship for Young Scientist to BW; Key Laboratory of Malignant Tumor Gene Regulation and Target Therapy of Guangdong Higher Education Institutes, Sun-Yat-Sen University (Grant No. KLB09001); Guangdong Provincial Clinical Research Center for Urological Diseases(2020B1111170006); and Key Laboratory of Malignant Tumor Molecular Mechanism and Translational Medicine of Guangzhou Bureau of Science and Information Technology (Grant No. 013-163).

## Conflict of Interest

The authors declare that the research was conducted in the absence of any commercial or financial relationships that could be construed as a potential conflict of interest.

## Publisher’s Note

All claims expressed in this article are solely those of the authors and do not necessarily represent those of their affiliated organizations, or those of the publisher, the editors and the reviewers. Any product that may be evaluated in this article, or claim that may be made by its manufacturer, is not guaranteed or endorsed by the publisher.

## Supplementary Material

Supplementary Figure 1 Cumulative overall survival curves of TCGA patients and cumulative recurrence-free survival curves of patients. **(A)** Kaplan-Meier survival estimates and log-rank tests were used to analyze the prognostic significance of TANs, which was characterized by the CIBERSORT method for bulk tumor transcriptomes in the TCGA database. *P*-values were determined using the log-rank test. Green lines, low group; Red lines, high group. **(B, C)** Kaplan-Meier recurrence-free survival curves for patients with UCB stratified by ${CD66b}_{\text{INT}}^{+}$ cells **(B)** and ${CD66b}_{\text{ST}}^{+}$ cells **(C)**. *P*-values were determined using the log-rank test. Black lines, low group; Red lines, high group.

Supplementary Figure 2 Kaplan-Meier overall survival curves stratified by histological grade. **(A)** Histological Low Grade (*n* = 145); **(B)** Histological High Grade (*n* = 92). *P*-values were determined using the log-rank test. Black lines, low group; Red lines, high group.

Supplementary Figure 3 TAN and TTN phenotypes. The indicated phenotype markers were assessed using flow cytometry, including the adhesin marker CD62L and chemokine receptors, CXCR1 and CXCR2. **(A)** Expression of the indicated markers on matched CD11b^+^CD66b^+^ PBNs and TANs. A single-cell suspension was obtained from freshly harvested tumor tissues and the peripheral blood of UCB patients. **(B)** The expression of the indicated markers was assessed on purified PBNs and TTNs. Autologous PBNs were purified from the PBMCs of healthy donors, and cultured with tumor cell line T24 supernatants, designed as tumor supernatant-treated neutrophils (TTNs). *P* < 0.05 was considered a significant difference (Student’s t-test, paired parametric test). **P* < 0.05; ***P* < 0.001; ****P* < 0.0001.

Supplementary Figure 4 Suppression of TTN on T cell immunity through programmed death-ligand 1 (PD-L1). **(A, B)** Consecutive sections were used for the immunohistochemical identification of stromal CD66b^+^ cells and exhausted T cell makers (PD1) in UCB tissues (n= 57). Representative examples of tumors with either low (left) or high (right) expression of neutrophil (CD66b), exhausted T cell makers (PD1) markers are shown. Scale bar: 100 μm. P < 0.05 was considered significant difference (Spearman’s rank correlation coefficient test). **(C–E)** In all experiments, autologous T cells were purified from PBMCs, stimulated with plate-bound anti-CD3/CD28 Abs, and cultured with tumor cell line T24 supernatant treated PBNs (TTNs) in the presence or absence of an anti-PD-L1 Ab at a 1:1 ratio for five days. Flow cytometric analysis of autologous T cell proliferation **(C)**, apoptosis **(D)**, and IFN-γ production **(E)** in the presence of PBNs, TTNs with or without an anti-PD-L1 Ab. Data shown are from one of six representative experiments in **(C–E)**. The numbers on the histograms or density plots represented the percentage of T cell proliferation or apoptosis, respectively **(C, D)**. Numbers in the quadrants indicate the percentage of cells in each quadrant **(E)**.

## Abbreviations

UCB, urothelial cell carcinoma of the bladder; TME, tumor microenvironment; TAN, tumor-associated neutrophils; MPO, myeloperoxidase; FFPE, formalin-fixed paraffin-embedded; RT-qPCR, real-time quantitative reverse transcription PCR; Ab, antibody; TSN, tumor supernatant; PBMCs, peripheral blood mononuclear cells; PBNs, peripheral blood neutrophils; TTN, TSN treated neutrophils; CFSE, carboxyfluorescein succinimidyl ester; PD-L1, programmed death ligand-1; PD-1, programmed death 1; TCGA, The Cancer Genome Atlas; BCG, bacillus Calmette-Guerin; INT, intra-tumoral; ST, stromal; OS, overall survival; RFS, recurrence-free survival; NMIBC, non-muscle-invasive bladder cancer; MIBC, muscle-invasive bladder cancer.
