# Supplementary figures and images for: Negative Effects of Stromal Neutrophils on T Cells Reduce Survival in Resectable Urothelial Carcinoma of the Bladder
Source: Front Immunol. 2022 Mar 21;13:827457. doi: 10.3389/fimmu.2022.827457 (PMC8978967; doi:10.3389/fimmu.2022.827457)

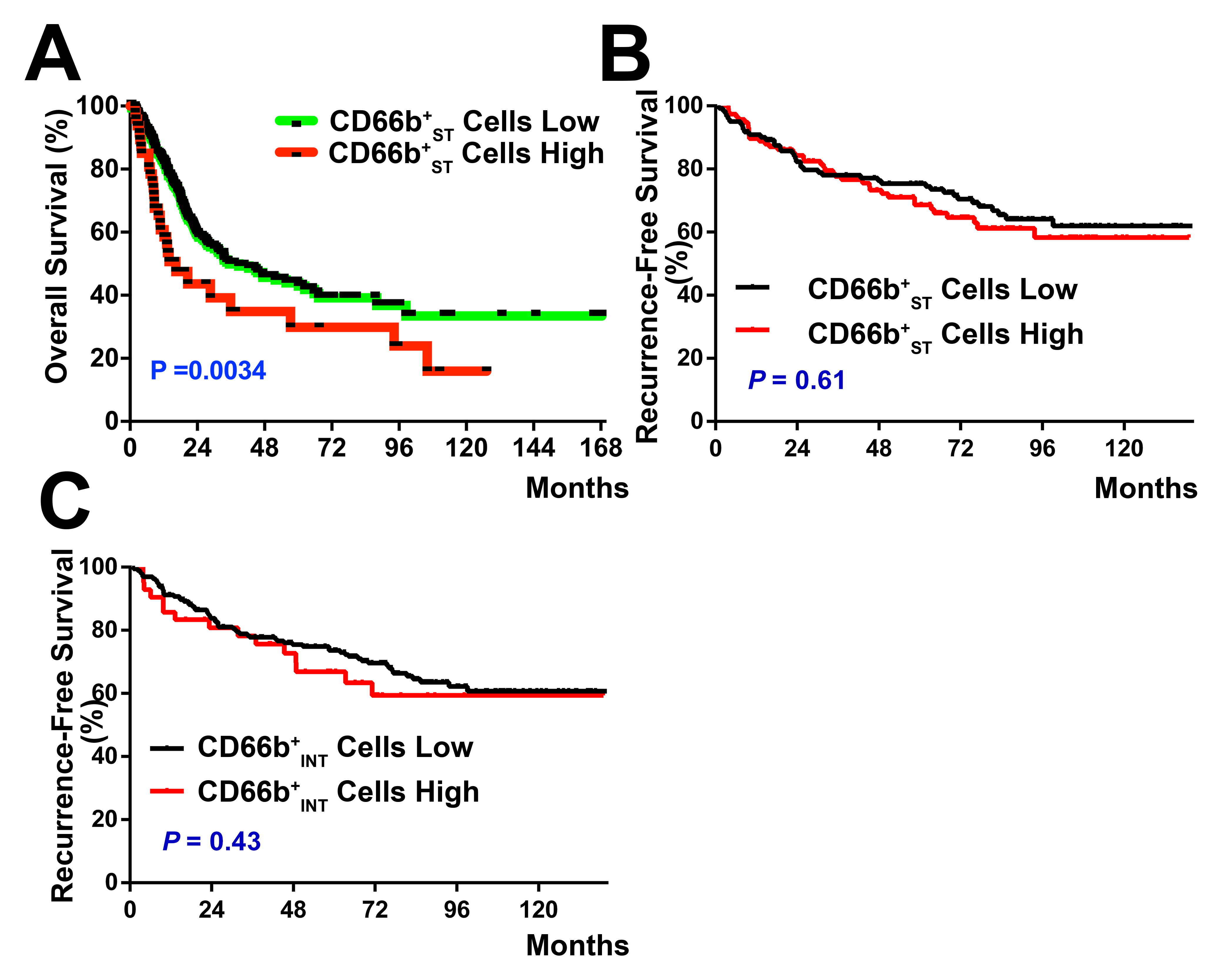

Supplement: Supplementary Figure 1 — Cumulative overall survival curves of TCGA patients and cumulative recurrence-free survival curves of patients. (A) Kaplan-Meier survival estimates and log-rank tests were used to analyze the prognostic significance of TANs, which was characterized by the CIBERSORT method for bulk tumor transcriptomes in the TCGA database. P-values were determined using the log-rank test. Green lines, low group; Red lines, high group. (B, C) Kaplan-Meier recurrence-free survival curves for patients with UCB stratified by CD66bINT+ cells (B) and CD66bST+ cells (C). P-values were determined using the log-rank test. Black lines, low group; Red lines, high group. [file Image_1.tif]

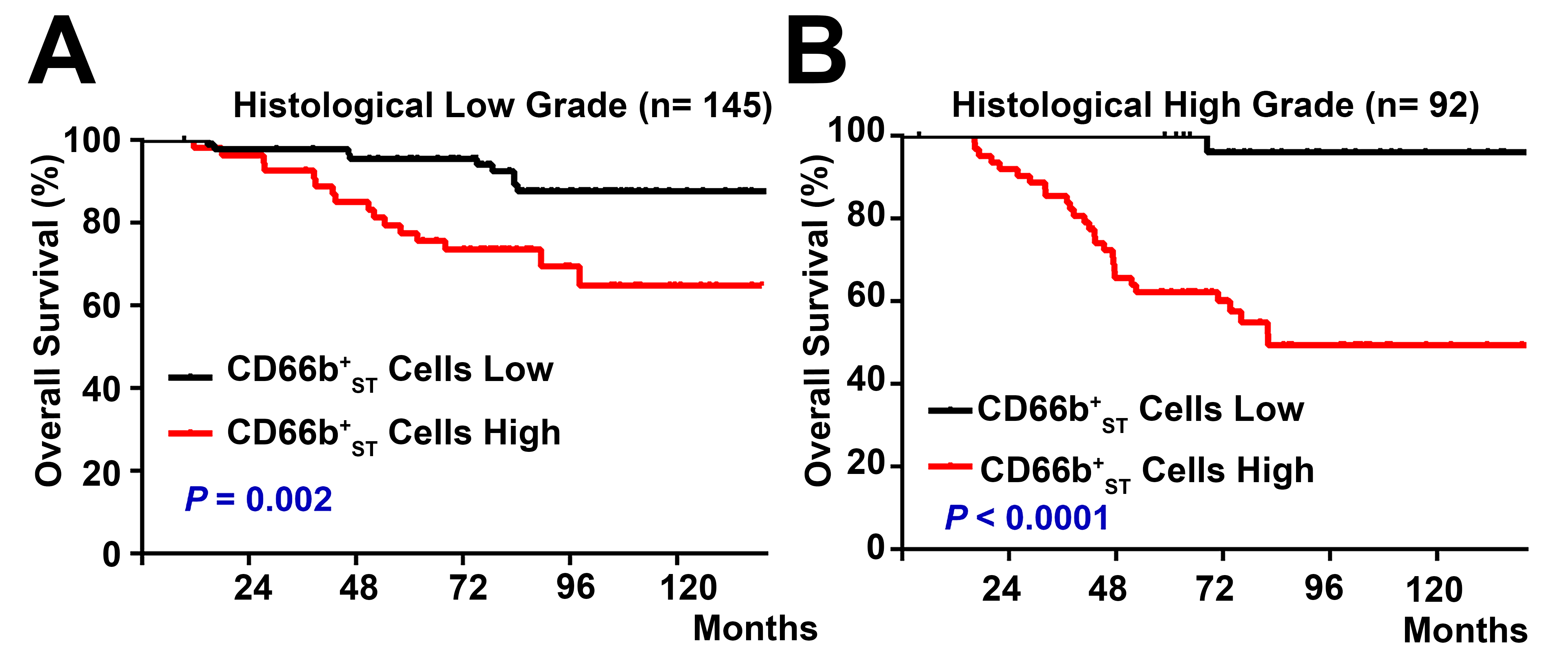

Supplement: Supplementary Figure 2 — Kaplan-Meier overall survival curves stratified by histological grade. (A) Histological Low Grade (n = 145); (B) Histological High Grade (n = 92). P-values were determined using the log-rank test. Black lines, low group; Red lines, high group. [file Image_2.tif]

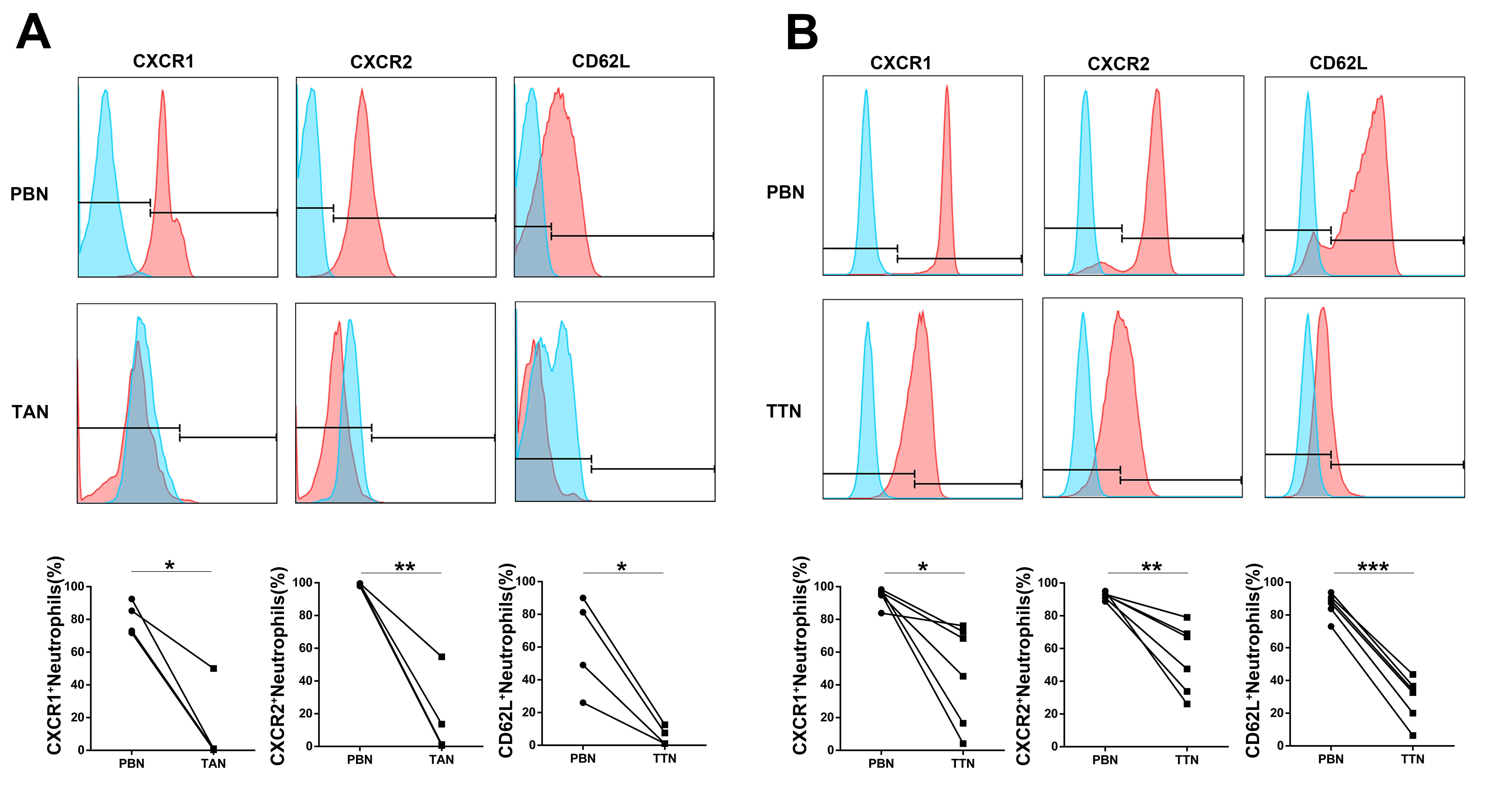

Supplement: Supplementary Figure 3 — TAN and TTN phenotypes. The indicated phenotype markers were assessed using flow cytometry, including the adhesin marker CD62L and chemokine receptors, CXCR1 and CXCR2. (A) Expression of the indicated markers on matched CD11b+CD66b+ PBNs and TANs. A single-cell suspension was obtained from freshly harvested tumor tissues and the peripheral blood of UCB patients. (B) The expression of the indicated markers was assessed on purified PBNs and TTNs. Autologous PBNs were purified from the PBMCs of healthy donors, and cultured with tumor cell line T24 supernatants, designed as tumor supernatant-treated neutrophils (TTNs). P < 0.05 was considered a significant difference (Student’s t-test, paired parametric test). *P < 0.05; **P < 0.001; ***P < 0.0001. [file Image_3.tif]

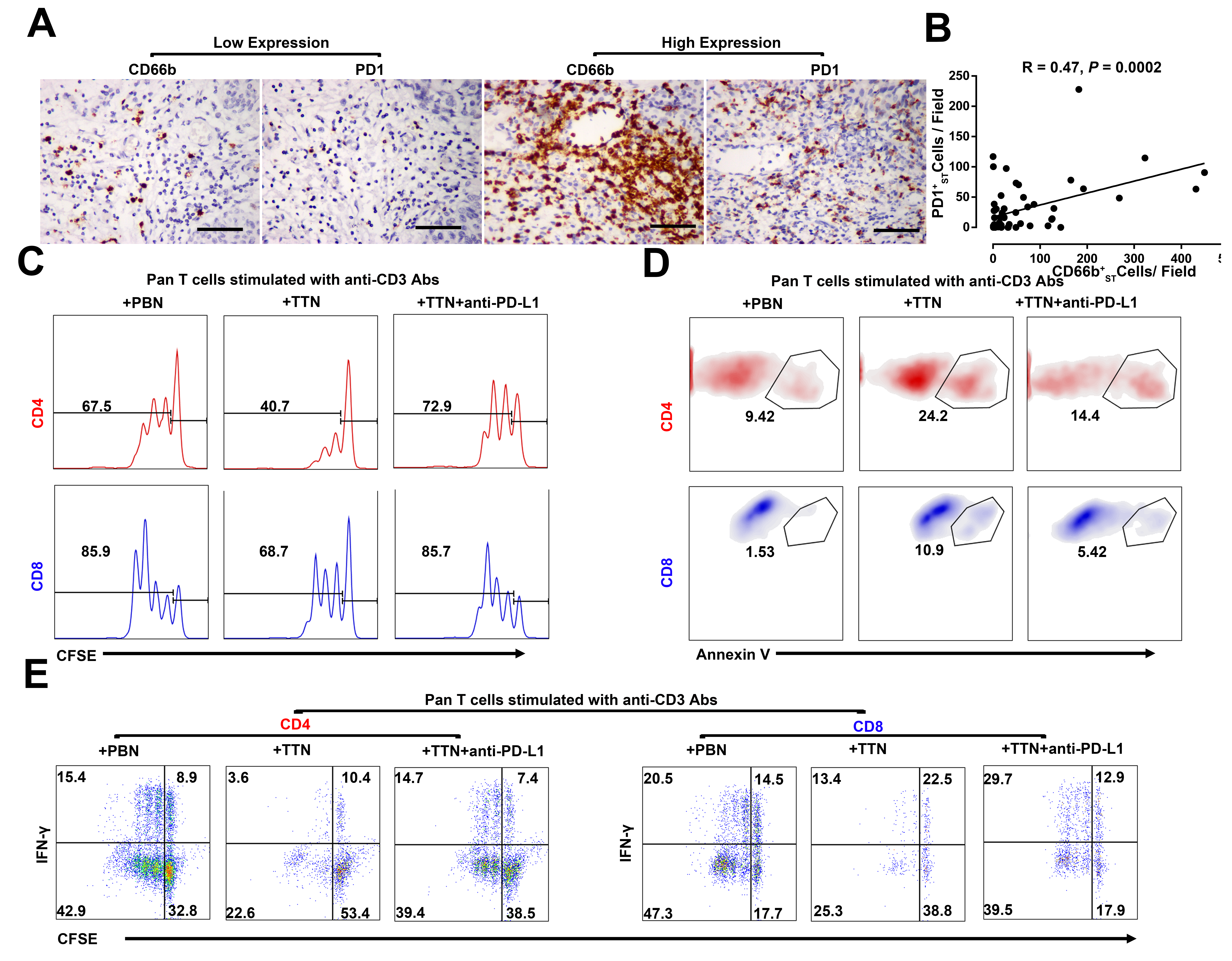

Supplement: Supplementary Figure 4 — Suppression of TTN on T cell immunity through programmed death-ligand 1 (PD-L1). (A, B) Consecutive sections were used for the immunohistochemical identification of stromal CD66b+ cells and exhausted T cell makers (PD1) in UCB tissues (n= 57). Representative examples of tumors with either low (left) or high (right) expression of neutrophil (CD66b), exhausted T cell makers (PD1) markers are shown. Scale bar: 100 μm. P < 0.05 was considered significant difference (Spearman’s rank correlation coefficient test). (C–E) In all experiments, autologous T cells were purified from PBMCs, stimulated with plate-bound anti-CD3/CD28 Abs, and cultured with tumor cell line T24 supernatant treated PBNs (TTNs) in the presence or absence of an anti-PD-L1 Ab at a 1:1 ratio for five days. Flow cytometric analysis of autologous T cell proliferation (C), apoptosis (D), and IFN-γ production (E) in the presence of PBNs, TTNs with or without an anti-PD-L1 Ab. Data shown are from one of six representative experiments in (C–E). The numbers on the histograms or density plots represented the percentage of T cell proliferation or apoptosis, respectively (C, D). Numbers in the quadrants indicate the percentage of cells in each quadrant (E). [file Image_4.tif]
