# Supplementary material for: Negative Effects of Stromal Neutrophils on T Cells Reduce Survival in Resectable Urothelial Carcinoma of the Bladder
Source: Front Immunol. 2022 Mar 21;13:827457. doi: 10.3389/fimmu.2022.827457 (PMC8978967; doi:10.3389/fimmu.2022.827457)
Supplement: Supplementary file 5 [file DataSheet_1.doc]

**Supplementary Table S1. Clinicopathological Characteristics of UCB (Group 1)**

| Variable | No. (%) |
| --- | --- |
| No. of patients | 237 |
| Age, years (median, range) | 60 (15–90) |
| Gender (male/ female) | 206/31 (86. 9/13.1) |
| Tumor size (≤ 3cm / > 3cm) | 188/49 (79.3/20.7) |
| Multifocality (unifocal/ multifocal) | 170/67 (71.7/28.3) |
| Tumor stage (Ta–T1/ T2–T4) | 171/66 (72.2/27.8) |
| Nodal status (N0/ N1–N2) | 227/10 (95.8/4.2) |
| Histological grade (Low/ High) | 145/92(61.2/38.8) |
| Follow–up, months (median, range) | 58.1(3.8–149) |

**Abbreviations:** UCB, urothelial cell carcinoma of the bladder.

Supplementary Table S2. Clinicopathological Characteristics of UCB (Group 2)

| **Variable** | **No. (%)** |
| --- | --- |
| No. of patients | 57 |
| Age, years (median, range) | 67 (22–87) |
| Gender (male/ female) | 48/9 (84. 2/15.8) |
| Tumor size (≤ 3cm / > 3cm) | 17/40 (29.8/70.2) |
| Multifocality (unifocal/ multifocal) | 34/23 (59.6/40.4) |
| Tumor stage (Ta–T1/ T2–T4) | 35/22 (61.4/38.6) |
| Nodal status (N0/ N1–N2) | 51/6 (89.5/10.5) |
| Histological grade (Low/ High) | 48/9(84.2/15.8) |

**Abbreviations:** UCB, urothelial cell carcinoma of the bladder.

**Supplementary Table S3. Key Resource** Table

| REAGENT or RESOURCE | SOURCE | IDENTIFIER |
| --- | --- | --- |
| **Antibodies** |  |  |
| Anti-human CD66b (Mouse monoclonal, Clone G10F5) | BD Pharmigen | Cat# 555723, RRID:AB_396066 |
| Anti-human Myeloperoxidase antibody (Rabbit monoclonal, Clone SP72) | Abcam | Cat# ab93665; RRID: AB_10562470 |
| Anti-human PD-L1 (Rabbit monoclonal, Clone 73-10) | Abcam | Cat# ab205921; RRID: AB_2687878 |
| Anti-human CD4 (Mouse monoclonal, Clone 4B12) | ThermoFisher | Cat# MS1528S0; RRID: AB_62559 |
| Anti-human CD8 (Mouse monoclonal, Clone C8/144B) | ThermoFisher | Cat# MS457S0; RRID: AB_61028 |
| Anti-mouse/human cleaved caspase 3 (Rabbit polyclonal) | Cell Signaling | Cat# 9661; RRID: AB_2341188 |
| Anti-human PD1 (Rabbit monoclonal, Clone D4W2) | Cell Signaling | Cat# 86163; RRID: AB_2728833 |
| FITC Anti-human CD62L clone DREG-56 | ThermoFisher | Cat# 11-0629-42;RRID:AB_10667774 |
| Brilliant Violet 510 Anti-human CD62L clone DREG-56 | Biolegend | Cat# 304844; RRID:AB_2617003 |
| PE Mouse Anti-human CD66b clone G10F5 | BD Pharmigen | Cat# 561650;RRID:AB_10894591 |
| PerCP/Cyanine5.5 Anti-human CD66b clone G10F5 | Biolegend | Cat# 305108; RRID:AB_2077855 |
| Brilliant Violet 510 Anti-human CD15 clone W6D3 | Biolegend | Cat# 323028; RRID:AB_2563400 |
| Brilliant Violet 605 Anti-human CD11b clone ICRF44 | Biolegend | Cat# 301332; RRID:AB_2562021 |
| Alexa Fluor® 488 Anti-human CD181 clone 8F1/CXCR1 | Biolegend | Cat# 320616;RRID:AB_2264875 |
| eFluor 450 Anti-human CD274 clone MIIH1 | ThermoFisher | Cat# 48-5983-42;RRID:AB_2574091 |
| APC Anti-human CD276 clone 7-517 | ThermoFisher | Cat# 17-2769-42;RRID:AB_2573190 |
| Brilliant Violet 650 Anti-human CD40 clone 5C3 | Biolegend | Cat# 334338;RRID:AB_2566209 |
| BV711 Mouse Anti-human CD134 clone ACT35 | BD Horizon | Cat# 563664;RRID:AB_2738359 |
| Alexa Fluor 700 Anti-human CD3 clone UCHT1 | ThermoFisher | Cat# 56-0038-42;RRID:AB_10597906 |
| APC/Fire™ 750 Anti-human CD4 clone RPA-T4 | Biolegend | Cat# 300560; RRID:AB_2629693 |
| CD8-PC7 | Beckman | Cat# 6607102; RRID:AB_10640793 |
| BUV395 Mouse Anti-human CD45 clone H130 | BD Horizon | Cat# 563792; RRID: AB_2744400 |
| eFluor 450 Anti-human IFN gamma clone 4S.B3 | ThermoFisher | Cat# 48-7319-42; RRID:AB_2043866 |
| **Critical Commercial Assays** |  |  |
| Dako REAL EnVision Detection System, Peroxidase/DAB+, Rabbit/Mouse | Dako | Cat#K5007 |
| Opal 4-Color Manual IHC Kit | PerkinElmer | Cat#NEL810001KT |
| Pan T Cell Isolation Kit, human | Miltenyi Biotec | Cat#130-096-535 |
| EasySep Human Neutrophil Enrichment Kit | Stemcell | Cat# 17957 |
| CellTrace™ CFSE Cell Proliferation Kit | Thermo Fisher | Cat# C34554 |
| Annexin V-APC/PI-PE Apoptosis Detection Kit | BestBio | Cat# BB-41033-2 |
| Fixable Viability Stain 510 | BD Horizon | Cat# 564406 |
| PrimeScript™ RT reagent Kit with gDNA Eraser | Takara | Cat# RR047A |
| LightCycler® 480 SYBR Green I Mast | Roche | Cat# 04707516001 |

**Supplementary Table S4**. Descriptive Statistics of Immunohistochemical Variables

| Variable* | Mean | SEM | Median | Range |
| --- | --- | --- | --- | --- |
| CD66b+INT cells | 3.2 | 0.72 | 0 | 0-69 |
| CD66b+ST cells | 26.06 | 2.68 | 5 | 0-226 |

**Abbreviations:** CD66b^+^_INT_ cells, CD66b^+^ cells in intratumoral regions; CD66b^+^_ST_ cells, CD66b^+^ cells in stromal regions. UCB, urothelial cell carcinoma of the bladder.

^*^ Number of cells per field (400×).

**Supplementary Table S5. Association of CD66b+INT TANs and CD66b^+^ST TANs to**

|  |  | **CD66b^+^_ST_ TANs** | | | |
| --- | --- | --- | --- | --- | --- |
| **Variable** |  | **Low** | **High** | **R** | **P** |
| No. of patients |  | 121 | 116 |  |  |
| Age, years |  |  |  | 0.139 | **0.033** |
| ≤ 60 |  | 71 | 52 |  |  |
| > 60 |  | 50 | 64 |  |  |
| Gender |  |  |  | –0.029 | 0.653 |
| Male |  | 104 | 102 |  |  |
| Female |  | 17 | 14 |  |  |
| Tumor size |  |  |  | 0.125 | 0.054 |
| ≤ 3cm |  | 102 | 86 |  |  |
| > 3cm |  | 19 | 30 |  |  |
| Multifocality |  |  |  | –0.034 | 0.607 |
| Unifocal |  | 85 | 85 |  |  |
| Multifocal |  | 36 | 31 |  |  |
| Tumor stage |  |  |  | 0.296 | **<0.0001** |
| Ta–T1 |  | 103 | 68 |  |  |
| T2–T4 |  | 18 | 48 |  |  |
| Nodal status |  |  |  | 0.13 | **0.045** |
| N0 |  | 119 | 108 |  |  |
| N1–N2 |  | 2 | 8 |  |  |
| Histological grade |  |  |  | 0.294 | **<0.0001** |
| Low |  | 91 | 54 |  |  |
| High |  | 30 | 62 |  |  |

**Clinical Parameters in UCB (n = 237)**

**Abbreviations:** CD66b^+^_ST_ TANs, CD66b^+^ tumor-associated neutrophils in stromal regions. UCB, urothelial cell carcinoma of the bladder.

Significant p-values are shown in bold font.

**Supplementary Table S6. Univariate and Multivariate Analysis of the Factors Associated with Overall Survival**

|  | | **Univariate** | | | |  | **Multivariate** | | | |
| --- | --- | --- | --- | --- | --- | --- | --- | --- | --- | --- |
| **Variable** | **HR** | | **95% CI** | **P** |  | | | **HR** | **95% CI** | **P** |
| Age, years (>60 / ≤60) | 3.53 | | 1.943–6.413 | **3.5×10^–5^** |  | | | 3.441 | 1.717–6.896 | **<0.0001** |
| Gender (female/male) | 0.694 | | 0.276–1.741 | 0.436 |  | | |  |  | NA |
| Tumor size (>3 cm / ≤3 cm) | 1.626 | | 0.896–2.951 | 0.11 |  | | |  |  | NA |
| Multifocality (Multifocal/Unifocal) | 0.922 | | 0.502–1.695 | 0.794 |  | | |  |  | NA |
| Tumor stage (T2–T4/Ta–T1) | 3.429 | | 2.009–5.854 | **6.3×10**^–6^ |  | | | 1.98 | 0.967–4.053 | **0.062** |
| Nodal status (N1–N2/N0) | 6.644 | | 2.933–15.051 | **5.7×10**^–6^ |  | | | 5.483 | 1.755–17.133 | **0.003** |
| Histological grade (High/Low) | 2.113 | | 1.236–3.612 | **0.006** |  | | | 1.284 | 0.62–2.656 | 0.501 |
| Combined CD66b^+^_ST_ Cells and CD4^+^_ST_ Cells |  | |  |  |  | | |  |  |  |
| Overall |  | |  |  |  | | |  |  |  |
| Ⅰvs Ⅲ | 2.783 | | 0.335–23.117 | 0.343 |  | | | 2.647 | 0.317–22.069 | 0.368 |
| Ⅱvs Ⅲ | 18.369 | | 2.424–139.2 | **0.005** |  | | | 11.662 | 1.512–89.977 | **0.018** |
| Ⅳvs Ⅲ | 11.088 | | 1.48–83.095 | **0.019** |  | | | 7.964 | 1.038–61.102 | **0.046** |

**Abbreviations:** I, CD66b^+^_ST_ Cells low CD4^+^_ST_ Cells low; II, CD66b^+^_ST_ Cells high CD4^+^_ST_ Cells low; III, CD66b^+^_ST_ Cells low CD4^+^_ST_ Cells high; IV, CD66b^+^_ST_ Cells high CD4^+^_ST_ Cells high. UCB, urothelial cell carcinoma of the bladder.

HR, hazard ratio; CI, confidence interval; NA, not applicable.

**NOTE:** Univariate and multivariate analysis. Cox proportional hazards regression model. Variables associated with survival by univariate analyses were adopted as covariates in the multivariate analyses. Significant P-values are shown in bold font.

HR > 1, risk for death increased; HR < 1, risk for death reduced.

|  | | **Univariate** | | | |  | **Multivariate** | | | |
| --- | --- | --- | --- | --- | --- | --- | --- | --- | --- | --- |
| **Variable** | **HR** | | **95% CI** | **P** |  | | | **HR** | **95% CI** | **P** |
| Age, years (>60 / ≤60) | 3.53 | | 1.943–6.413 | **3.5×10^–5^** |  | | | 3.29 | 1.715–6.314 | **<0.0001** |
| Gender (female/male) | 0.694 | | 0.276–1.741 | 0.436 |  | | |  |  | NA |
| Tumor size (>3 cm / ≤3 cm) | 1.626 | | 0.896–2.951 | 0.11 |  | | |  |  | NA |
| Multifocality (Multifocal/Unifocal) | 0.922 | | 0.502–1.695 | 0.794 |  | | |  |  | NA |
| Tumor stage (T2–T4/Ta–T1) | 3.429 | | 2.009–5.854 | **6.3×10**^–6^ |  | | | 1.977 | 1.024–3.815 | **0.042** |
| Nodal status (N1–N2/N0) | 6.644 | | 2.933–15.051 | **5.7×10**^–6^ |  | | | 3.579 | 1.281–10.002 | **0.015** |
| Histological grade (High/Low) | 2.113 | | 1.236–3.612 | **0.006** |  | | | 0.95 | 0.492–1.834 | 0.879 |
| Combined CD66b^+^_ST_ Cells and CD8^+^_ST_ Cells |  | |  |  |  | | |  |  |  |
| Overall |  | |  |  |  | | |  |  |  |
| Ⅰvs Ⅲ | 3.468 | | 0.439–27.376 | 0.238 |  | | | 3.143 | 0.395–25.027 | 0.279 |
| Ⅱvs Ⅲ | 9.845 | | 1.247–77.714 | **0.03** |  | | | 8.361 | 1.051–66.543 | **0.045** |
| Ⅳvs Ⅲ | 17.309 | | 2.359–127 | **0.005** |  | | | 10.754 | 1.431–80.793 | **0.021** |

**Supplementary Table S7. Univariate and Multivariate Analysis of the Factors Associated with Overall Survival**

**Abbreviations:** I, CD66b^+^_ST_ Cells low CD8^+^_ST_ Cells low; II, CD66b^+^_ST_ Cells high CD8^+^_ST_ Cells low; III, CD66b^+^_ST_ Cells low CD8^+^_ST_ Cells high; IV, CD66b^+^_ST_ Cells high CD8^+^_ST_ Cells high. UCB, urothelial cell carcinoma of the bladder.

HR, hazard ratio; CI, confidence interval; NA, not applicable.

**NOTE:** Univariate and multivariate analysis. Cox proportional hazards regression model. Variables associated with survival by univariate analyses were adopted as covariates in multivariate analyses. Significant P-values are shown in bold font.

HR > 1, risk for death increased; HR < 1, risk for death reduce
