# Supplementary material for: Negative Effects of Stromal Neutrophils on T Cells Reduce Survival in Resectable Urothelial Carcinoma of the Bladder
Source: Front Immunol. 2022 Mar 21;13:827457. doi: 10.3389/fimmu.2022.827457 (PMC8978967; doi:10.3389/fimmu.2022.827457)
Supplement: Supplementary file 6 [file DataSheet_2.docx]

**Supplementary Materials**

**Immunohistochemistry and immunofluorescence**

FFPE tissues from study Groups 1 and 2 were cut into 5-μm sections, and subsequently processed for immunohistochemistry as previously described. After standard antigen retrieval, the samples were incubated with human-specific anti-CD66b (BD Biosciences, San Jose, CA, USA, 1:500 dilution), anti-CD4 (Thermo Fisher Scientific, Waltham, MA, USA, 1:500 dilution), anti-CD8 (Thermo Fisher Scientific, Waltham, MA, USA, 1:200 dilution), anti-PD1 (CST, Danvers, MA, USA, 1:500 dilution), anti-Cleaved Caspase 3 (CST, Danvers, MA, USA, 1:500 dilution) or control antibodies (Abcam, Cambridge, UK). The adjacent sections were developed with peroxidase-conjugated secondary antibodies and stained with peroxidase and 3,3′-diaminobenzidine tetrahydrochloride in an Envision System (Dako). Sections were then counterstained with hematoxylin (Zhongshan Bio-Tech Co., Zhongshan, China) and mounted with a nonaqueous mounting medium.

Multiplexed fluorescent immunohistochemistry was performed by the Tyramide Signal Amplification (TSA) method using an Opal IHC kit (PerkinElmer, Waltham, MA), and FFPE tissue sections were processed according to the standard IHC protocol described above. The primary antibodies were anti-human CD66b, CD4, CD8, MPO, and PD-L1. Using the Opal method, two or three human primary antibodies were sequentially applied to a single slide, overnight followed by an incubation with appropriate peroxidase-conjugated secondary antibodies (Dako, K5007) for 30 min at room temperature. Detection was performed with an incubation with FITC-, Cy3-, or Cy5-labeled Tyramide (PerkinElmer, 1:50). Microwave heating was performed for primary antigen unmasking and antibody removal after each fluorescent labeling. The slides were counterstained with DAPI (Sigma Aldrich, 1:2000) for 1 min. The Zeiss LSM710 system with the ZEN software (Zeiss, Oberkochen, Germany) was used to capture the images of the stained slides and perform an image analysis.

**Preparation of a single-cell suspension from UCB tissue**

Surgically removed fresh UCB tissues were trimmed, sliced into 1 – 2 mm^3^ pieces, and digested in RPMI 1640 supplemented with 1% penicillin-streptomycin, collagenase type I and IV (170 mg/L = 45–60 U/mL), collagenase type II (56 mg/L = 15 – 20 U/mL), DNase I (25 mg/L), and elastase (25 mg/L) (all from Worthington Biochemical) for 1 h at 37°C with agitation. Dissociated cells were filtered through a 70-µM nylon cell strainer (BD Falcon). After the filtration the red blood cells lysed using 1x Red Blood Cell (RBC) Lysis Buffer (CWBIO; Peking). The remaining cells were washed and resuspended in medium supplemented with 1% heat-inactivated FCS for FACS analysis.

**Tumor cell line and TSN preparation**

The T24 human bladder cancer cell line was obtained from the American Type Culture Collection. The cells were tested for mycoplasma contamination using a single-step polymerase chain reaction (PCR) method and maintained in a complete medium composed of RPMI 1640 supplemented with 10% fetal bovine serum. Tumor culture supernatant (TSN) were prepared by plating 5 × 10^6^ tumor cells in 10 mL complete medium in 100-mm dishes. After 72 h, TSN was collected, centrifuged, aliquoted, and stored at -80°C.

**Neutrophil and lymphocyte isolation**

Peripheral blood mononuclear cells (PBMCs) from buffy coats derived from healthy donors were isolated by Ficoll density gradient centrifugation. CD3^+^ T cells from PBMCs were purified using a Pan T cell isolation kit (Miltenyi Biotec, Bergisch Gladbach, Germany). After the lysis of red blood cells, peripheral blood neutrophils (PBNs) were further enriched by positively removing contaminating cells using an EasySep Human Neutrophil Enrichment Kit (Stemcell Technologies). The purity of isolated PBNs was evaluated by flow cytometry upon staining for the neutrophils/myeloid markers, CD66b, CD15, CD62L, and CD11b in cells used in functional assays. The sorted cells were not used unless their viability was determined > 90% and their purity was determined > 95%. In some experiments, the PBNs in RPMI 1640 containing fetal bovine serum were cultured in the presence of 20% TSN for 24 h to obtain TSN-treated neutrophils (TTNs) to mimic TANs in UCB tissues.

**Neutrophil-T cell co-culture system**

PBNs and matched TTNs were co-cultured with T cells (responders) from the autologous peripheral blood of healthy donors to study the function of neutrophils on T cell proliferation, activation, and apoptosis.

Purified T cells (responders) were labeled with 5 µM of the fluorescent dye 5, 6-carboxyfluorescein diacetate succinimidyl ester (CFSE) (ThermoFisher), according to the manufacturer’s instructions. CFSE-labeled T cells were stimulated with plate-bound anti-human CD3 Ab (Biolegend, clone: OKT3). To coat the 96 U-bottom-well plates, anti-CD3 Ab (1 µg/mL) were added to 100 µL of PBS/well and incubated overnight at 4°C. The wells were washed twice with PBS before the addition of cells. CFSE-labeled responders were mixed with either PBNs or TTNs at a 1:1 ratio and co-cultured in CD3-coated plates for three days in the complete cell culture media. In several experiments, blocking Abs against PD-L1 (clone: IT2.2, Biolegend) were added to the co-cultures of TTNs and activated T cells at a concentration of 5µg/mL. The blocking Ab was also coated on the plate with CD3 Abs and presented in a TTNs/T cell co-culture for three days.

To evaluate proliferation and activation, CFSE and IFN-γ signals were analyzed by ﬂow cytometry on gated CD4^+^ or CD8^+^ T cells. In other experiments, for the apoptosis assay, T cells were first stained with an APC-Cy7 anti-human CD4 antibody and BV650 anti-human CD8 antibody, washed, and then stained with an Annexin V FITC-PI staining kit (BestBio; Shanghai) according to the manufacturer’s instruction.

**Flow cytometry**

Flow cytometric analysis was performed according to standard protocols. Cell suspensions from the tissues and peripheral blood of UCB patients, as well as *in vitro* cultured neutrophils or T cells were stained with fluorochrome-conjugated antibodies (Additional file 1: Table S3). Matched-isotype Abs were used as controls. To exclude dead cells from the analysis, the cells were stained with Fixable Viability Dye BV510 (BD Biosciences). For intracellular staining, the cells were fixed and intracellular staining with BD Perm/Wash™ Buffer (BD Biosciences) and stained with BV421-anti-human IFN-γ (Biolegend, clone: 4S.B3) for 45 min at RT. The data were acquired using the BD FACSCalibur or BD LSRFortessaTM (BD Bioscience) flow cytometers and analyzed using FlowJo software (TreeStar Inc.).
